# The Pharmacokinetic Profile of a New Gastroresistant Capsule Preparation of Eicosapentaenoic Acid as the Free Fatty Acid

**DOI:** 10.1155/2015/360825

**Published:** 2015-08-03

**Authors:** Eleonora Scaioli, Carla Cardamone, Elisa Liverani, Alessandra Munarini, Mark A. Hull, Andrea Belluzzi

**Affiliations:** ^1^Gastroenterology Unit, Department of Medical and Surgical Sciences, Sant'Orsola-Malpighi University Hospital, 40138 Bologna, Italy; ^2^Center for Applied Biomedical Research (C.R.B.A.), Sant'Orsola-Malpighi University Hospital, 40138 Bologna, Italy; ^3^Section of Molecular Gastroenterology, Leeds Institute of Biomedical & Clinical Sciences, St James's University Hospital, Leeds LS1 3EX, UK

## Abstract

Supplementation with n-3 polyunsaturated fatty acids (n-3 PUFAs) may be beneficial for patients with inflammatory bowel diseases (IBD). In this study we analyzed the pharmacokinetic profile of eicosapentaenoic acid (EPA), as the free fatty acid (FFA), in an enteric-coated preparation, in 10 ulcerative colitis (UC) and 10 Crohn's disease (CD) patients and 15 healthy volunteers (HV). Subjects received 2 g daily of EPA-FFA for 8 weeks. Plasma phospholipid and red blood cell (RBC) membrane fatty acid content were measured by gas chromatography-mass spectrometry. There was a rapid incorporation of EPA into plasma phospholipids by 2 weeks and a slower, but highly consistent, incorporation into RBC membranes (4% total fatty acid content; coefficient of variation 10–16%). There was a concomitant reduction in relative n-6 PUFA content. Elongation and desaturation of EPA into docosahexaenoic acid (DHA) via docosapentaenoic acid (DPA) were apparent and DHA content also increased in membranes. EPA-FFA is well tolerated and no difference in the pharmacokinetic profile of n-3 PUFA incorporation was detected between IBD patients and HV. Our data support the concept that EPA can be considered the “universal donor” with respect to key n-3 PUFAs and that this enteric-coated formulation allows long term treatment with a high level of compliance.

## 1. Introduction

The major natural dietary source of long-chain n-3 polyunsaturated fatty acids (n-3 PUFAs) is cold-water, oily fish, which can be consumed safely in large quantities. However, despite the large scale and safe consumption reported in Eskimos [1], this dietary habit only occurs in a small proportion of individuals in the industrialised and developing world. Fish oil can be administered in pharmaceutical form for many different therapeutic purposes such as chronic inflammatory diseases [2–4], treatment of hyperlipidaemia [5], and after myocardial infarction [6].

Oral administration of fish oil containing the two main bioactive components C20:5n3 eicosapentaenoic acid (EPA) and C22:6n3 docosahexaenoic acid (DHA) can replace C18:2n6 linoleic acid (LA) and C20:4n6 arachidonic acid (AA) in a time- and dose-dependent manner in plasma and cellular phospholipid membranes [2].

Plasma n-3 PUFA level is the easiest marker of EPA and DHA intake for measuring compliance in taking supplements as various fish oil preparations. However, it is established that the plasma phospholipid fatty acid profile may change within a period of hours, depending on the type and timing of food intake [7]. Analysis of red blood cell (RBC) membrane n-3 PUFA content is understood to be a more reliable measure [8]. The relatively long half-life of the RBC (120 days) provides a more stable measure of the incorporation of fatty acids into cellular phospholipid membranes [9]. The Omega-3 index (the combined percentage EPA, docosapentaenoic acid-DPA, and DHA content in RBC phospholipid membranes) can reach ≥8% with achievable n-3 PUFA intake [10].

It is clear that although fish oil has no serious toxicity, minor adverse events (AEs) such as dysgeusia, flatulence, pyrosis, halitosis, belching, and abdominal discomfort are common and may limit compliance [11, 12]. Enteric coating of the capsules containing the fish oil may help to minimise upper gastrointestinal effects.

There are conflicting data on the comparative bioavailability and adverse event profiles of n-3 PUFAs conjugated to a glycerol chain, as an ethyl ester conjugate and as the free fatty acid [13], but direct comparison of the three forms suggests that bioavailability is highest with the free fatty acid and lowest with the ethyl ester [14].

We decided to test compliance with and the pharmacokinetic profile of a new preparation containing EPA in the free fatty acid (FFA) form presented in gastroresistant capsules.

Since supplementation with n-3 polyunsaturated fatty acids (n-3 PUFAs) has been claimed to be beneficial in patients with chronic inflammatory conditions, even if results were controversial [3, 4, 15–17], we enrolled a group of patients with inflammatory bowel diseases (IBD), ulcerative colitis (UC), and Crohn's disease (CD). The patients were in stable clinical remission (≥3 months), our main purpose being to supplement patients with presumed good absorption characteristics, whilst avoiding inclusion of those with diarrhoea. A control group of healthy volunteers (HV) was recruited from medical students.

## 2. Materials and Methods

### 2.1. Study Design

The study was conducted in accordance with the Declaration of Helsinki and approved by the Ethics Committee and the Clinical Board of Sant'Orsola-Malpighi Hospital, Bologna. Written, informed consent was obtained from 20 IBD patients (10 CD and 10 UC), who were in stable clinical remission according to routine clinical scores (Crohn's Disease Activity Index—CDAI < 150 and Simple Clinical Colitis Activity Index—SCCAI = 0) [18, 19] for at least 3 months, attending the outpatient clinic, and 15 HV recruited among medical students.

Exclusion criteria were previous allergy/intolerance to n-3 PUFAs, existing use of n-3 PUFA-containing supplements, previous bowel resection more than 1 metre, pregnancy, or desire to become pregnant. During the study, subjects were asked not to change their dietary habits.

All subjects received 2 g EPA-FFA daily as two 500 mg capsules (ALFA) twice a day with food for 8 weeks.

Venous blood was taken in an EDTA tube after an overnight fast at baseline (time zero) and at 2, 4, and 8 weeks to obtain plasma and RBCs. Routine screening blood tests, including blood count, erythrocyte sedimentation rate, serum creatinine, and liver function tests, were performed at baseline and the end of the study.

Adherence to the dosing regimen was evaluated at each clinic visit by interview and by capsule count. Subjects were considered adherent if they used at least 80% of the capsules between each visit, with no interruption of supplementation for more than 14 consecutive days. At each visit, diary cards were checked for adverse events (AEs).

### 2.2. Fatty Acid Analysis

The detailed description of the phospholipid fatty acid assay has been previously published [20].

In brief, total lipids were extracted from 2 mL of plasma and from 2 mL of packed RBCs. Phospholipids were separated from the total lipid fraction by one-dimensional thin-layer chromatography [21].

Red blood cell membranes were then processed following the procedure of Popp-Snijders et al. [22]. Red cell membrane lipids were extracted according to Dodge and Phillips [23], using a 2 : 1 (v : v) mixture of chloroform and methanol containing 0.01% butylated hydroxytoluene (2,6 di-*tert*-bury-p-cresol, Sigma) as antioxidant. Samples were then stored under nitrogen at −20°C for a maximum of 2 weeks prior to fatty acid analysis.

Fatty acids were transmethylated using 1 N potassium hydroxide in methanol and boron trifluoride in 14% methanol for 10 minutes at 80°C [24]. Fatty acid methyl-esters were then extracted in hexane, resuspended in 100 *μ*L of benzene, and analysed by gas chromatography-mass spectrometry. Individual fatty acid methyl-esters were identified by comparison with authentic standards (Sigma). Heptadecanoic acid (17 : 0) was used as an internal calibrating standard (1 mg/mL in benzene) and the results are expressed as the percentage of total phospholipid fatty acids [25].

The following PUFAs were analysed: LA, AA, EPA, C22:5n3 docosapentaenoic acid (DPA), and DHA.

### 2.3. Statistical Methods

The geometric mean and standard deviation (SD) of the mean are provided for each fatty acid. The plasma and RBC phospholipid fatty acid content of each fatty acid in each group over time were compared by the Kruskal-Wallis test or the Wilcoxon rank test. Absolute intraindividual changes in PUFA content were analysed using the one-sample *t*-test and data are quoted as the mean and 95% confidence interval. Variability of the change in PUFA content over time between individuals was described using the percentage (%) coefficient of variation.

Differences between groups were compared using the Mann-Whitney (MW) *U* test.

In all cases, statistical significance was assumed if *P* ≤ 0.05.

## 3. Results

### 3.1. Subjects

Characteristics of the study subjects are summarized in Table 1. There were no statistically significant differences between subjects at baseline.

### 3.2. Baseline PUFA Profile in Plasma Phospholipids and RBC Membranes

Baseline plasma and RBC PUFA profiles in IBD patients and HV are noted in Tables 2 and 3. As expected, in individuals consuming a “western” diet, the n-6 PUFA content was in greater than 10-fold excess of the relative n-3 PUFA content.

The plasma n-3 PUFA content in IBD patients was significantly higher than in HVs (MW: *P* < 0.01; Table 2). However, the RBC n-6 PUFA content in IBD patients was significantly higher than HV (*P* < 0.01; Table 3). Both of these observations are consistent with previous reports [9, 26].

There was no significant difference in baseline PUFA profiles between CD and UC patients in clinical remission. CD and UC site involvement did not predict the baseline plasma or RBC PUFA profile (data not shown).

The mean baseline Omega-3 indices in HV and IBD patients were 4.1% and 3.7%, respectively.

### 3.3. Changes in Plasma and RBC PUFA Content during EPA-FFA Treatment

Tables 2 and 3 detail the changes in the plasma and RBC membrane content of n-3 and n-6 PUFAs during 8-week treatment with EPA-FFA.

**Figure 1:**
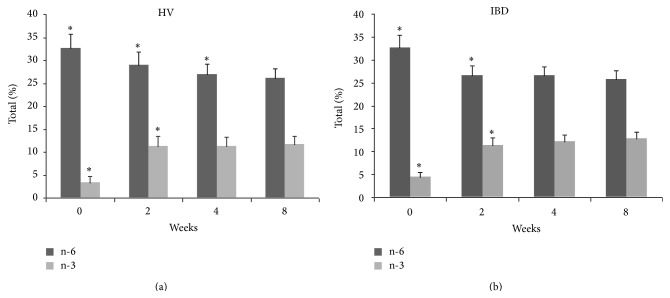
Plasma polyunsaturated fatty acids (PUFAs) profile during eicosapentaenoic acid-free fatty acids (EPA-FFA) treatment in healthy volunteers (HV) and inflammatory bowel disease (IBD) patients. Data are the mean (column) and standard deviation (bar) of the total n-6 PUFAs (linoleic acid + arachidonic acid; dark shade) and n-3 PUFAs (eicosapentaenoic acid + docosapentaenoic acid + docosahexaenoic acid; light shade); % of total PUFA content at each time point. ^*∗*^ *P* < 0.001.

**Figure 2:**
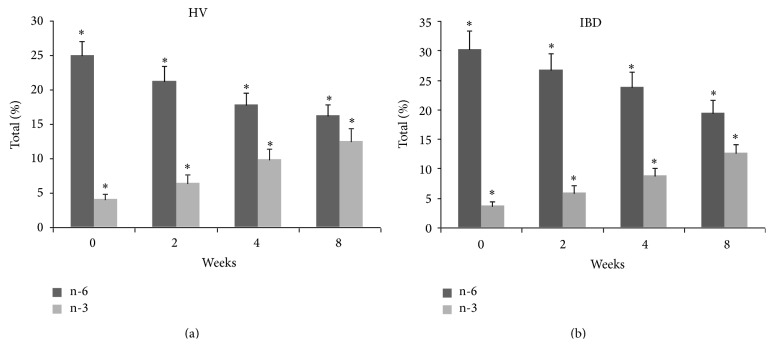
Red blood cell membrane polyunsaturated fatty acids (PUFAs) profile during eicosapentaenoic acid-free fatty acids (EPA-FFA) treatment in healthy volunteers (HV) and inflammatory bowel disease (IBD) patients. Data are the mean (column) and standard deviation (bar) of the total n-6 PUFAs (linoleic acid + arachidonic acid; dark shade) and n-3 PUFAs (eicosapentaenoic acid + docosapentaenoic acid + docosahexaenoic acid; light shade); % of total PUFA content at each time point. ^*∗*^ *P* < 0.001.

**Figure 3:**
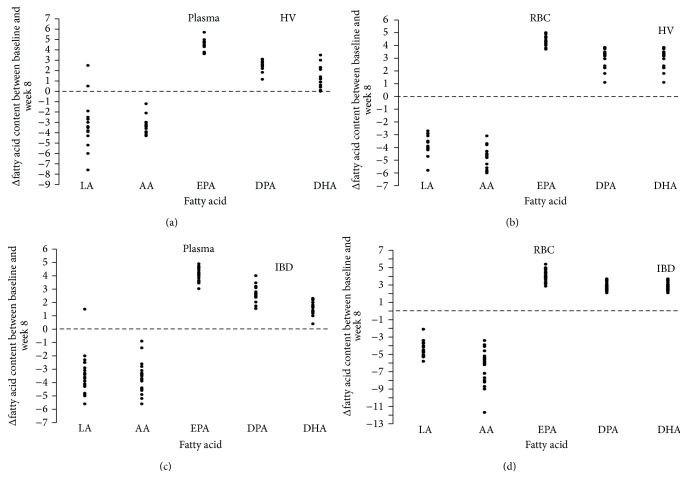
Interindividual variability in the absolute change in polyunsaturated fatty acids (PUFAs) content of plasma and red blood cell (RBC) membrane from baseline to week 8 in healthy volunteers (HV) and inflammatory bowel disease (IBD) patients. LA: linoleic acid; AA: arachidonic acid; EPA: eicosapentaenoic acid; DPA: docosapentaenoic acid; DHA: docosahexaenoic acid.

The change in plasma PUFA profile occurred rapidly, being evident after only 2 weeks of supplementation with % content reaching steady-state between the 4th and the 8th week (Table 2 and Figure 1). By contrast, changes in the RBC membrane PUFA profile occurred more slowly and were progressive up to 8 weeks (Table 3 and Figure 2). In both phospholipid compartments, there was a statistically significant increase in incorporation of all n-3 PUFAs and a concomitant decrease in n-6 PUFA content (in all cases *P* < 0.001; Kruskal-Wallis test). Individual changes in fatty acid content in plasma and RBC membrane were similar in both HV and IBD groups with little interindividual variability in EPA and DPA incorporation but larger variability in the reduction of n-6 PUFAs and DHA (Tables 2 and 3, Figure 3). There was no significant correlation between the change in plasma PUFA content in the first two weeks and the change in RBC PUFA content at 8 weeks (data not shown) suggesting that early changes in plasma FA content do not predict longer-term tissue PUFA incorporation during EPA-FFA treatment.

The mean Omega-3 indices in HV and IBD patients were 12.5% and 12.6%, respectively, which is consistent with previous studies [10].

There were no significant differences in PUFA content between HV and IBD patients, suggesting that there is comparable incorporation (and, by inference, absorption) in both groups. There was also no significant difference in the PUFA profile change between CD and UC patients overall or on the basis of the site of IBD involvement (data not shown).

At the end of the treatment, the n-6 PUFA/n-3 PUFA ratio was much lower in both HV and IBD patients being 3 : 1 and 2 : 1 in plasma and RBC membranes.

### 3.4. Compliance and Side Effects

EPA-FFA 2 g daily was well tolerated and there was no participant drop out. All subjects satisfied the criteria for compliance (i.e., less than 20% of capsules returned and no reported interruption for more than 14 days). Some minor/mild side effects were reported and these are summarised in Table 4. The most commonly reported side effects were mild gastrointestinal disturbance, which did not result in treatment cessation. Importantly, IBD patients did not report high incidence of diarrhoea. No serious adverse events were reported.

## 4. Discussion

To our knowledge, this is the first pharmacokinetic study to evaluate the incorporation of n-3 PUFAs into RBCs with an EPA-only preparation over a period of more than 4 weeks in humans.

Treatment with EPA-FFA has been evaluated in several randomised trials and has proved to be safe and well tolerated [27, 28]. However, the kinetics of EPA incorporation and changes in the content of other PUFAs during EPA-FFA ingestion have not been studied systematically. Herein, we demonstrate efficient and consistent EPA incorporation into plasma phospholipids and RBC membranes. Consistent with the concept that EPA is the “universal donor” [29], EPA-FFA treatment led to increased relative content of both EPA and DHA. Importantly, early changes in plasma phospholipid PUFA content did not predict the eventual PUFA profile in RBCs at 8 weeks. Therefore, we can confirm that the plasma PUFA level is not a reliable biomarker of longer-term tissue incorporation.

We and others have demonstrated that the FFA form of n-3 PUFAs provides the most favourable PK profile in comparison with ethyl ester and triglyceride preparations. Improved absorption of the FFA is believed to be due to its ability to cross the intestinal wall directly, without the requirement for lipase activity [5, 14, 30].

We have demonstrated that EPA-FFA is rapidly and consistently incorporated into plasma phospholipids and RBC membranes in healthy volunteers as well as IBD patients in remission. Our data show that the absorption and the metabolism of n-3 PUFAs are similar in patients with IBD and HVs, with a comparable level of omega-3 absorption and incorporation. This is a fundamental requirement when considering long term treatment with omega-3 PUFAs. The benefit of n-3 PUFAs in patients with IBD is still a matter of debate. Recent reviews and meta-analyses still leave open the question as to the efficacy of n-3 PUFA supplementation in IBD patients, and the results of the trials over the years are not in favour of chronic omega-3 supplementation [4, 17, 31, 32]. Nevertheless a recent paper by Costea et al. [33] reopens the debate, showing that, in a subgroup of children with a specific gene pattern who developed CD, there was a profound imbalance between n-6/n-3 PUFAs, suggesting that supplementation with n-3 PUFAs would be beneficial.

Our previous experience in this field is in favour of supplementation with n-3 PUFAs and we would wish to have available the most efficacious and well tolerated omega-3 preparation for our patients.

We tested ALFA EPA-free fatty acid capsules in a group of IBD patients in stable clinical remission and, encouragingly, we found that data on incorporation and compliance were comparable to those of healthy volunteers.

After just 2 months' treatment, the total relative n-3 PUFA content (the Omega-3 index) was greater than 12% in both HV and IBD patients, which compares favourably with that attained after mixed EPA/DHA supplementation [34] and which exceeds the recognized threshold Omega-3 index after n-3 PUFA treatment [10].

DHA content also increased in membranes, implying elongation and desaturation of EPA into DHA via DPA. The increase in RBC DHA content that we observed is similar to that seen after daily dosing for 8 weeks with six capsules containing a total of 1,296 mg EPA and 864 mg DHA [34]. It is recognized that DHA content after supplementation with n-3 PUFA (EPA+DHA) exceeds that associated with EPA alone, which is consistent with our data [34, 35]. Supplementation with DHA alone would be important to compare the effects of the two different n-3 PUFAs in humans.

In keeping with experience with EPA-FFA in randomized trials [27, 28], only a small number of mild side effects were registered which may be explained by the formulation of EPA-FFA in gastroresistant capsules.

## 5. Conclusions

Our data confirm that administration of EPA-FFA results in rapid tissue EPA incorporation. EPA can be quickly converted into DHA via DPA so that EPA can be considered the “universal donor” of n-3 PUFAs. As the EPA-FFA gastroresistant capsules employed in the study enable n-3 PUFA supplementation in high doses with a small number of capsules, this formulation overcomes the poor compliance observed with mixed PUFA fish oil derivatives that require dosing with large numbers of capsules.

## Figures and Tables

**Table 1:** Subject characteristics.

Characteristic°	Crohn's disease (*N* = 10)	Ulcerative colitis (*N* = 10)	Healthy volunteers (*N* = 15)
Age (years) mean ± SD	39 ± 11	34 ± 10	28 ± 8
Male *n* (%)	6 (60)	4 (40)	5 (33.3)
Current smoker *n* (%)	3 (30)	1 (10)	4 (26.6)
Duration of disease (months)			
Mean ± SD	68 ± 32	65 ± 28	—
Previous intestinal resection *n* (%)	2 (20)	0 (0)	0 (0)
CDAI^*^			
Median (range)	82 (38–102)	/	/
SCCAI^*^			
Median (range)	/	0 (0)	/
Site of involvement *n* (%)			
Ileum	4 (40)	/	/
Ileum and colon	3 (30)	/	/
Colon	3 (30)	/	/
Pancolitis	/	3 (30)	/
Left-sided colitis	/	7 (70)	/
Ongoing drug therapy *n* (%)			
Immunosuppressive agents	4 (40)	2 (20)	/
Mesalamine	6 (60)	8 (80)	

^*∗*^CDAI: Crohn's disease activity index; SCCAI: simple clinical colitis activity index; °*P* = ns.

**Table 2:** Fatty acid content of plasma phospholipids in healthy volunteers (HV) and inflammatory bowel disease (IBD) patients.

	Fatty acid (% of total)		Baseline	2 weeks	4 weeks	8 weeks	Change in fatty acid content from baseline to week 8^*^	%CV
HV	n-6 PUFAs	C18:2 LA	19.1 ± 2.2^**^	17.3 ± 1.3	16.4 ± 1.3	15.9 ± 1.1	−3.2 (−4.6 to −1.9)^***^	75.2
		C20:4 AA	13.6 ± 0.9	11.6 ± 1.7	10.5 ± 0.9	10.3 ± 0.8	−3.2 (−3.7 to −2.8)	25.0
	n-3 PUFAs	C20:5 EPA	0.3 ± 0.2	4 ± 0.8	4.5 ± 1	4.6 ± 0.6	4.3 (4.0–4.7)	14.5
		C22:5 DPA	0.3 ± 0.3	2.6 ± 0.7	2.7 ± 0.5	2.8 ± 0.5	2.5 (2.2 to 2.8)	19.8
		C22:6 DHA	2.7 ± 0.8	4.6 ± 0.7	4.0 ± 0.5	4.2 ± 0.7	1.5 (0.9–2.1)	70.9

IBD	n-6 PUFAs	C18:2 LA	18.5 ± 1.6	16 ± 1.1	15.7 ± 1	15.2 ± 1	−3.4 (−4.1 to −2.6)	45.2
		C20:4 AA	14.2 ± 1	10.7 ± 0.9	11 ± 0.8	10.6 ± 0.9	−3.6 (−4.1 to −3.1)	32.0
	n-3 PUFAs	C20:5 EPA	0.6 ± 0.2°	4.2 ± 0.7	4.8 ± 0.6	4.7 ± 0.5	4.1 (3.8 to 4.3)	12.3
		C22:5 DPA	0.5 ± 0.2°	2.6 ± 0.5	2.9 ± 0.4	3.1 ± 0.6	2.6 (2.3 to 2.9)	24.0
		C22:6 DHA	3.3 ± 0.6°	4.5 ± 0.5	4.4 ± 0.5	4.9 ± 0.4	1.6 (1.4 to 1.8)	32.1

^*∗∗*^Values are mean ± SD.

°*P* < 0.01 for the difference in individual n-3 PUFA content between IBD patients and HV.

^*^Absolute change in % fatty acid content.

^***^Data are the mean and 95% confidence interval. All *P* < 0.01.

PUFAs: polyunsaturated fatty acids.

LA: linoleic acid.

AA: arachidonic acid.

EPA: eicosapentaenoic acid.

DPA: docosapentaenoic acid.

DHA: docosahexaenoic acid.

CV: coefficient of variation.

**Table 3:** Fatty acid content of red blood cell membranes in healthy volunteers (HV) and inflammatory bowel disease (IBD) patients.

	Fatty acid		Baseline	2 weeks	4 weeks	8 weeks	Change in fatty acid content from baseline to week 8^*^	%CV
HV	n-6 PUFAs	C18:2 LA	10.5 ± 1.4^**^	9.1 ± 1	7.7 ± 1	6.5 ± 1	−4.0 (−4.5 to −3.5)^***^	24.0
		C20:4 AA	14.5 ± 0.7	12.2 ± 1.2	10.1 ± 0.7	9.7 ± 0.6	−4.8 (−5.2 to −4.3)	17.9
	n-3 PUFAs	C20:5 EPA	0.3 ± 0.1	1.7 ± 0.4	4 ± 0.5	4.5 ± 0.4	4.3 (4.0 to 4.5)	9.8
		C22:5 DPA	0.8 ± 0.2	1.4 ± 0.4	2.2 ± 0.4	3.8 ± 0.7	2.9 (2.5 to 3.4)	27.3
		C22:6 DHA	3 ± 0.5	3.3 ± 0.5	3.6 ± 0.7	4.2 ± 0.8	1.2 (0.7 to 1.7)	69.4

IBD	n-6 PUFAs	C18:2 LA	12.2 ± 1°	11.1 ± 0.8	9.7 ± 0.8	7.8 ± 0.8	−4.4 (−4.8 to −3.9)	20.4
		C20:4 AA	18 ± 2.1°	15.7 ± 1.9	14.1 ± 1.7	11.6 ± 1.5	−6.5 (−7.4 to −5.5)	31.0
	n-3 PUFAs	C20:5 EPA	0.2 ± 0.1	1.1 ± 0.3	2.1 ± 0.4	4.3 ± 0.7	4.1 (3.7 to 4.4)	16.3
		C22:5 DPA	0.4 ± 0.1	1.1 ± 0.6	2.2 ± 0.5	3.3 ± 0.4	2.8 (2.6 to 3.0)	15.5
		C22:6 DHA	3.1 ± 0.5	3.7 ± 0.4	4.5 ± 0.4	5 ± 0.4	1.9 (1.6 to 2.2)	33.5

^*∗∗*^Values are mean ± SD.

°*P* < 0.01 for the difference in individual n-3 PUFA content between IBD patients and HV.

^*^Absolute change in % fatty acid content.

^***^Data are the mean and 95% confidence interval. All *P* ≤ 0.01.

PUFAs: polyunsaturated fatty acids.

LA: linoleic acid.

AA: arachidonic acid.

EPA: eicosapentaenoic acid.

DPA: docosapentaenoic acid.

DHA: docosahexaenoic acid.

CV: coefficient of variation.

**Table 4:** Adverse event profile of eicosapentaenoic acid-free fatty acid (EPA-FFA).

	*n* (%)	Frequency
HV		
Diarrhoea	1 (6.7)	4
Nausea	2 (13.3)	4
Abdominal pain/distension	2 (13.3)	4
Epigastric discomfort	2 (13.3)	3
Headache	1 (6.7)	3
IBD patients		
Diarrhoea	3 (15)	5
Nausea	2 (10)	4
Abdominal pain/distension	3 (15)	5
Epigastric discomfort	3 (15)	3
Headache	2 (10)	4

Frequency is the absolute number of times given adverse events occurred.

HV: healthy volunteers.

IBD: inflammatory bowel disease.

## Abbreviations

PUFAs: Polyunsaturated fatty acids
LA: Linoleic acid
AA: Arachidonic acid
EPA: Eicosapentaenoic acid
DPA: Docosapentaenoic acid
DHA: Docosahexaenoic acid
FFA: Free fatty acid
IBD: Inflammatory bowel diseases
UC: Ulcerative colitis
CD: Crohn's disease.

## References

[1] Sinclair H. M. (1953). The diet of Canadian Indians and eskimos. *Proceedings of the Nutrition Society*.

[2] Calder P. C. (2013). Omega-3 polyunsaturated fatty acids and inflammatory processes: Nutrition or pharmacology?. *British Journal of Clinical Pharmacology*.

[3] Wall R., Ross R. P., Fitzgerald G. F., Stanton C. (2010). Fatty acids from fish: the anti-inflammatory potential of long-chain omega-3 fatty acids. *Nutrition Reviews*.

[4] Cabré E., Mañosa M., Gassull M. A. (2012). Omega-3 fatty acids and inflammatory bowel diseases-a systematic review. *British Journal of Nutrition*.

[5] Davidson M. H., Johnson J., Rooney M. W., Kyle M. L., Kling D. F. (2012). A novel omega-3 free fatty acid formulation has dramatically improved bioavailability during a low-fat diet compared with omega-3-acid ethyl esters: the ECLIPSE (Epanova compared to Lovaza in a pharmacokinetic single-dose evaluation) study. *Journal of Clinical Lipidology*.

[6] GISSI-Prevenzione Investigators (Gruppo Italiano per lo Studio della Sopravvivenza nell'Infarto miocardico) (1999). Dietary supplementation with n-3 polyunsaturated fatty acids and vitamin E after myocardial infarction: results of the GISSI-Prevenzione trial. *The Lancet*.

[7] von Schacky C., Weber P. C. (1985). Metabolism and effects on platelet function of the purified eicosapentaenoic and docosahexaenoic acids in humans. *The Journal of Clinical Investigation*.

[8] von Schacky C., Fischer S., Weber P. C. (1985). Long-term effects of dietary marine omega-3 fatty acids upon plasma and cellular lipids, platelet function, and eicosanoid formation in humans. *Journal of Clinical Investigation*.

[9] Belluzzi A., Brignola C., Campieri M. (1994). Effects of new fish oil derivative on fatty acid phospholipid-membrane pattern in a group of Crohn's disease patients. *Digestive Diseases and Sciences*.

[10] Saravanan P., Davidson N. C., Schmidt E. B., Calder P. C. (2010). Cardiovascular effects of marine omega-3 fatty acids. *The Lancet*.

[11] O'Connor G. T., Malenka D. I., Olmstead E. M., Johnson P. S., Hennekens C. H. (1992). A meta-analysis of randomized trials of fish oil in prevention of restenosis following coronary angioplasty. *American Journal of Preventive Medicine*.

[12] Olsen S. F., Sorensen J. D., Secher N. J. (1992). Randomised controlled trial of effect of fish-oil supplementation on pregnancy duration. *The Lancet*.

[13] Schuchardt J. P., Hahn A. (2013). Bioavailability of long-chain omega-3 fatty acids. *Prostaglandins Leukotrienes & Essential Fatty Acids*.

[14] Lawson L. D., Hughes B. G. (1988). Human absorption of fish oil fatty acids as triacylglycerols, free acids, or ethyl esters. *Biochemical and Biophysical Research Communications*.

[15] Belluzzi A., Brignola C., Campieri M., Pera A., Boschi S., Miglioli M. (1996). Effect of an enteric-coated fish-oil preparation on relapses in Crohn's disease. *The New England Journal of Medicine*.

[16] Feagan B. G., Sandborn W. J., Mittmann U. (2008). Omega-3 free fatty acids for the maintenance of remission in crohn disease: the EPIC randomized controlled trials. *Journal of the American Medical Association*.

[17] Turner D., Shah P. S., Steinhart A. H., Zlotkin S., Griffiths A. M. (2011). Maintenance of remission in inflammatory bowel disease using omega-3 fatty acids (fish oil): a systematic review and meta-analyses. *Inflammatory Bowel Diseases*.

[18] Best W. R., Becktel J. M., Singleton J. W., Kern F. (1976). Development of a Crohn's disease activity index. National cooperative Crohn's disease study. *Gastroenterology*.

[19] Walmsley R. S., Ayres R. C. S., Pounder R. E., Allan R. N. (1998). A simple clinical colitis activity index. *Gut*.

[20] Folch J., Lees M., Stanley G. H. S. (1957). A simple method for the isolation and purification of total lipides from animal tissues. *The Journal of Biological Chemistry*.

[21] Schlierf G., Wood P. (1965). Quantitative determination of plasma free fatty acids and triglycerides by thin-layer chromatography. *Journal of Lipid Research*.

[22] Popp-Snijders C., Schouten J. A., de Jong A. P., van der Veen E. A. (1984). Effect of dietary cod-liver oil on the lipid composition of human erythrocyte membranes. *Scandinavian Journal of Clinical and Laboratory Investigation*.

[23] Dodge J. T., Phillips G. B. (1967). Composition of phospholipids and of phospholipid fatty acids and aldehydes in human red cells.. *Journal of Lipid Research*.

[24] Morrison W. R., Smith L. M. (1964). Preparation of fatty acids methyl esters and dimethylacetals. *Journal of lipid research*.

[25] Latorre A., Rigol A., Lacorte S., Barceló D. (2003). Comparison of gas chromatography-mass spectrometry and liquid chromatography-mass spectrometry for the determination of fatty and resin acids in paper mill process waters. *Journal of Chromatography A*.

[26] Esteve-Comas M., Núñez M. C., Fernández-Bañares F. (1993). Abnormal plasma polyunsaturated fatty acid pattern in non-active inflammatory bowel disease. *Gut*.

[27] West N. J., Clark S. K., Phillips R. K. S. (2010). Eicosapentaenoic acid reduces rectal polyp number and size in familial adenomatous polyposis. *Gut*.

[28] Cockbain A. J., Volpato M., Race A. D. (2014). Anticolorectal cancer activity of the omega-3 polyunsaturated fatty acid eicosapentaenoic acid. *Gut*.

[29] Fini L., Piazzi G., Ceccarelli C. (2010). Highly purified eicosapentaenoic acid as free fatty acids strongly suppresses polyps in Apc^Min/+^ mice. *Clinical Cancer Research*.

[30] El Boustani S., Colette C., Monnier L., Descomps B., Crastes De Paulet A., Mendy F. (1987). Enteral absorption in man of eicosapentaenoic acid in different chemical forms. *Lipids*.

[31] Swan K., Allen P. J. (2013). Omega-3 fatty acid for the treatment and remission of Crohn's disease. *Journal of Complementary and Integrative Medicine*.

[32] Turner D., Zlotkin S. H., Shah P. S., Griffiths A. M. (2007). Omega 3 fatty acids (fish oil) for maintenance of remission in Crohn's disease. *Cochrane Database of Systematic Reviews*.

[33] Costea I., MacK D. R., Lemaitre R. N. (2014). Interactions between the dietary polyunsaturated fatty acid ratio and genetic factors determine susceptibility to pediatric Crohn's disease. *Gastroenterology*.

[34] Cao J., Schwichtenberg K. A., Hanson N. Q., Tsai M. Y. (2006). Incorporation and clearance of omega-3 fatty acids in erythrocyte membranes and plasma phospholipids. *Clinical Chemistry*.

[35] Katan M. B., Deslypere J. P., van Birgelen A. P. J. M., Penders M., Zegwaard M. (1997). Kinetics of the incorporation of dietary fatty acids into serum cholesteryl esters, erythrocyte membranes, and adipose tissue: an 18-month controlled study. *Journal of Lipid Research*.

